# SALA-LSTM: a novel high-precision maritime radar target detection method based on deep learning

**DOI:** 10.1038/s41598-023-39348-3

**Published:** 2023-07-26

**Authors:** Jingang Wang, Songbin Li

**Affiliations:** 1 Institute of Acoustics, Chinese Academy of Sciences, Beijing, 100190 China; 2 University of Chinese Academy of Sciences, Beijing, 100049 China

**Keywords:** Electrical and electronic engineering, Network models

## Abstract

Radar detection of maritime targets plays an important role in marine environment monitoring. For civil maritime detection in the areas of inshore coastal, pulse-compression radar is universally used owing to its low cost. The complex sea clutter in the practical application will greatly affect the received radar echoes. Due to the inability to accurately describe the differences in characteristics between sea clutter and maritime targets, the detection performance of methods based on mathematical derivation is not satisfactory in actual deployment. Recently, neural-based methods have made strides in many pattern recognition tasks, such as computer vision and natural language processing. The sophisticated deep neural models can be applied to different downstream tasks due to their powerful learning ability. Inspired by this idea, we propose a maritime radar target detection method in sea clutter based on deep learning. To better model the sequence correlation of radar echoes, we propose a Self-Adaption Local Augmented Long Short-Term Memory (SALA-LSTM) structure. The proposed SALA-LSTM integrates adaptive convolution into vanilla LSTM cells, which not only maintains the inherent overall sequence modeling ability of vanilla LSTM, but also strengthens its ability to perceive the correlation on a small scale in the local scope. Based on SALA-LSTM and other neural structures, we propose a radar target detection network. A measured dataset containing different typical scenarios is utilized to evaluate the detection probability and false alarm rate. The detection performance of our proposed network is superior to that of the existing methods.

## Introduction

Radar has characteristics of all-weather and all-time, and can realize long-distance detection and tracking for targets-of-interest. Compared with visual light, radar is less affected by light, climate and other interference conditions. Radar-based maritime target detection has become a popular paradigm^1^. Radar can obtain relevant information about the maritime targets by receiving the scattering echo of the sea surface. In addition to the target signal, the echo signal of the radar also contains sea clutter^2^ and other interference signals, which brings difficulty to the detection of maritime targets. When the target detection algorithm processes this sudden extremely strong echo, it will cause a large false alarm. The sea clutter is affected by the wave height, wind speed, duration, sea conditions, etc. of the sea wave itself, as well as radar parameters such as grazing angle, pulse repetition frequency, range resolution unit size, and antenna polarization, so it is difficult to describe the sea clutter with a single statistical model. This brings great difficulty to radar maritime target detection under a sea clutter background.

Early radar target detection methods were generally based on the basic idea of CFAR (Constant False-Alarm Rate), such as CA-CFAR^3^ and GO-CFAR^4^. Although these methods based on simple mathematical calculations have high efficiency, its detection performance is relatively poor. With the development of machine learning, methods based on hand-crafted features have been proposed^5–11^. The commonly used paradigm is to design one or several features by time-frequency domain analysis, and utilized these features to classify sea clutter and maritime targets. However, due to the inability to ideally describe the differences in characteristics between sea clutter and maritime targets, the detection performance of methods based on mathematical derivation is not satisfactory. These methods cannot meet the requirements of a low false alarm rate in practical applications. In recent years, neural-based methods have made strides in many pattern recognition tasks, such as computer vision and natural language processing. The sophisticated deep neural models can be applied to different downstream tasks due to their powerful learning ability. Inspired by this idea, we propose a maritime radar target detection method in sea clutter based on deep learning. Our goal is to use this data-driven approach to enable the model to automatically learn effective features. However, considering the actual application scenarios, we still face the following three challenges when applying deep learning technology to the radar detection of maritime targets.

The first is the requirement of complex sea clutter on the detection robustness of radar target detection network. The characteristics of sea clutter under different sea states are complex and changeable, and this complex sea clutter will cause a great interference to the radar echo. Especially in the background of strong sea clutter, the signal-to-clutter ratio (SCR) of radar reflection of maritime targets is further reduced. This requires the detection algorithm to reach strong robustness. Otherwise, the strong sea clutter will be misjudged as maritime targets, which will increase the false alarm rate (FAR). The radar detection system will be disturbed. The second is the requirement of diverse maritime targets on the detection generalization of radar target detection network. In practical scenarios, it is unfeasible to forecast the maritime targets that the radar system will encounter during continual monitoring operations. The echo characteristics of some possible small targets, such as sampans and fishing boats, are different from those of some large targets, such as ocean-going cargo ships and vessels, which brings difficulties to detection. The detection model must be able to adapt to maritime targets of different sizes and achieve satisfactory generalization detection performance. The third is the requirement for a controllable low false alarm rate of radar target detection network in practical application scenarios. The core problem of radar target detection is to determine whether there are maritime targets in the current received radar echo. Compared with the common two-class problem, the false alarm rate is very important for radar target detection. At present, the radar system deployed in sea surveillance cannot reach a satisfactory early warning performance because of the serious false alarm. Only by controlling the false alarm rate can the radar target detection technology based on deep learning be truly put into practice.

How to effectively address the above issues is an important basis for the successful application of deep learning technology in radar target detection. To this end, this paper intends to construct a deep network from the following basic ideas. First of all, the detection robustness can be improved by integrating the feature information from global and local perspectives. From the global perspective, the model can perceive the overall power level of sea clutter. From the local perspective, the model can mine the weak target signal submerged in the sea clutter. Secondly, adaptive network structure selection based on current radar echo characteristics can improve detection generalization. Due to the diversity of sea surface targets and the large range of size changes, using the fixed coding network structure after training to extract features of targets with different echo characteristics may affect the generalization detection performance. It may be a possible solution to improve the generalization detection capability so that the coding network structure can be selected adaptively according to the current echo. Finally, the detection model should also have a controllable false alarm, to reduce the false alarm of the radar monitoring system under certain circumstances. It is a feasible idea to reduce the false alarm rate by using the classification threshold of the neural network.

Based on the above basic ideas, we propose a Self-Adaption Local-Augmented Long Short-Term Memory (SALA-LSTM) structure in this paper. SALA-LSTM can perform feature correlation extraction of currently received radar echoes from both global and local perspectives, thus avoiding misjudgment of sea clutter or targets from a single perspective. Besides, it can adjust the network parameters adaptively according to the current echo input. Based on SALA-LSTM and other neural structures, we propose a maritime radar target detection method. The contributions can be summarized as follows:

1. To model the radar echo sequence, we propose a self-adaption local augmented LSTM (SALA-LSTM) structure. SALA-LSTM introduces an adaptive convolution layer to replace the multiplication operation in the vanilla LSTM. By this module, we can obtain an effective feature representation for pulse-compression radar echoes.
2. This paper proposes a radar target detection method. The proposed method first employs a signal reconstruction module to convert the original radar echo into a form that is easy to be processed by the subsequent neural network. Then, SALA-LSTM is used to construct an effective feature representation. By progressive prediction module, the obtained features are then dimensionally reduced layer by layer to extract important information and finally converted into a binary classification result. This paradigm is of certain reference value to the relevant researchers.
3. Based on the deployed X-band pulse-compression radar, we collected measured radar echoes and constructed a maritime target dataset to evaluate the detection performance of our proposed method. Experimental results demonstrate that the detection performance of the proposed method exceeds the existing methods.

## Background and related works

Sea clutter is defined as the sea surface backscattered echo received when the radar electromagnetic wave irradiates the sea surface^12^. It is not only affected by environmental factors (such as wind speed, wave height, wave crest, etc.), but also by radar parameters (such as frequency, polarization, beam incidence angle, etc.), thus the types are extremely complex. Scholars have carried out a lot of research on the statistical modeling of sea clutter, which mainly includes two aspects. On the one hand, the real data of sea clutter under different marine environments are obtained by actual measurement^13–15^, and the statistical characteristics are obtained on this basis for direct use; On the other hand, based on statistical theory, different statistical models^16,17^ are studied, analyzed and simulated.

Radar target detection in the sea clutter background has a wide range of applications and important practical value in military and civilian scenes, and is still developing. The classical radar target detection method is CFAR^18–20^. The basic idea is to estimate the noise level in the current radar echo by using the units around the unit to be measured (CUT) to dynamically calculate the threshold. For specific implementation, the power of a fixed number of cells near the current cell is generally calculated. If the power of the current cell exceeds the power of the surrounding cells, it is determined that there is a target in the current cell. However, this kind of algorithm is usually difficult to achieve the given false alarm rate in the low SCR (Signal-to-Clutter Ratio) scene under the sea clutter interference, which restricts the improvement of radar system detection performance. For the target detection in sea clutter, some researchers have proposed a series of methods based on statistical features^5–11^.

Shui et al.^5^ used the three characteristics of the receiving vector at the resolution unit, the relative amplitude of the Doppler amplitude spectrum, the relative Doppler peak height, and the relative entropy to distinguish whether the echo contains an effective target. Based on the observed multi-polarization channel echo, Xu et al.^6^ extracted three polarization characteristics, relative surface scattering power, relative volume scattering power and relative dihedral scattering power, and achieved a detection effect superior to that in Ref.^5^. Yan et al.^7^ proposed a detector using mean spectral radius (MSR). They defined MSR as a specific linear spectral statistic indicating data correlation, and proved through experiments that MSR is an effective feature to distinguish target echo and sea clutter. Besides, the velocity changes of the target and sea clutter can also be used as detection features^8–11^. For example, Chen et al.^8^ studied the micro-Doppler effect and proposed a micro-Doppler signal extraction algorithm based on short-time fractional Fourier transform (STFRFT).

The above traditional methods are difficult to achieve satisfactory detection performance in practical applications. Hence, researchers^21–28^ began to explore the method based on deep learning. For example, Chen et al.^21^ took the Doppler spectrum and radar echo amplitude as the original input, and used a dual-channel convolutional neural network and a false alarm controllable classifier to extract features and obtain prediction results. On this basis, Qu et al.^27^ proposed a more sophisticated network called STDN. It consists of ResNet backbone and asymmetric convolution layer. The enhanced CNN structure^27^ takes full advantage of the latent information in time-frequency inputs. Wan et al.^28^ proposed a method for detecting small maritime targets based on sequence feature extraction. In their method, the instantaneous phase, Doppler spectrum entropy, and short-time Fourier transform marginal spectrum are combined to train a Bi-LSTM model to conduct target detection. Jing et al.^22^ introduced the idea of comparative learning into radar sea surface target detection. By constructing positive and negative sample pairs of sea clutter effective targets, the network can adapt to more complex sea clutter interference. Graph neural network is another possible strategy. Su et al.^23^ utilize graph structure data to define the detection units and to represent the temporal and spatial information of detection units. On this basis, they propose a detection method based on a graph convolution network.

## Method

In this paper, we propose a method for radar detection of maritime targets based on self-adaption local augmented LSTM (SALA-LSTM). This echo sequence of pulse-compression radar as the model input and conducts a prediction of whether the currently received radar echo contains any maritime target. The overall framework of our proposed method is illustrated in section “Overall structure”. The main neural-based modules are described in sections “Signal reconstruction module”, “Self-adaption local augmented LSTM”, “Progressive prediction module” respectively.

**Figure 1:**
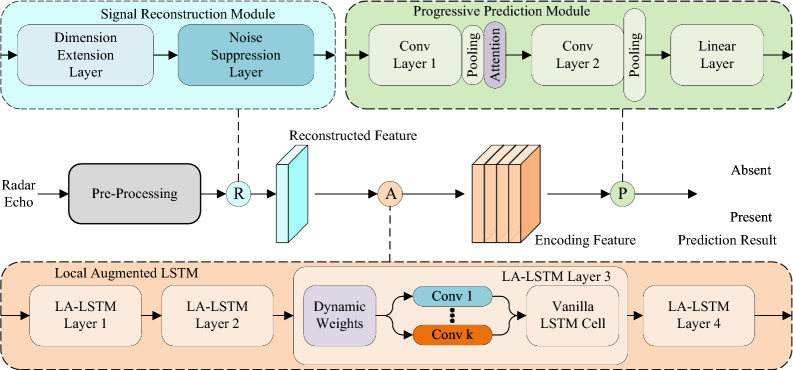
The overall structure of our proposed method.

### Overall structure

As shown in Fig. 1, the overall structure of the proposed method mainly consists of pre-processing, signal reconstruction module, SALA-LSTM, and progressive prediction module. Through these four modules, we can obtain the prediction results, absent or present. For the original radar echo, matched filtering based on frequency domain is firstly performed. Then, we use the space-time combination window to obtain the windowed signal. Assuming that $X_t$ represents radar echo signal received at a time *t*:

1 $$\begin{aligned} X_t=\left\{ x_1,x_2,\ldots ,x_n\right\} \end{aligned}$$

where *n* is the observation length. We pack all sampling points of a windowed sample which cascading *m* radar echoes together into a input matrix *F*:

2 $$\begin{aligned} F=\left\{ X_1,X_2,\ldots ,X_m\right\} \end{aligned}$$

Amplitude normalization is also needed for fast convergence during network training.

Then, we utilize the signal reconstruction module to expand the dimension of the original signal while suppressing the noise therein. The specific implementation for noise suppression is to assign a small weight to the noise part of the input echo. Next, we propose a sequence encoding structure named local adaptive augmented LSTM (LA-LSTM) to conduct effective feature encoding for radar echoes. On the basis of vanilla LSTM, this structure additionally enhances the dynamic perception of local feature correlations. The obtained encoding feature will finally be fed into the progressive prediction module. The module employs a convolution structure to achieve dimensionality reduction layer by layer and gradually extract key information. A linear layer is employed to obtain the two-class prediction results.

### Signal reconstruction module

**Figure 2:**
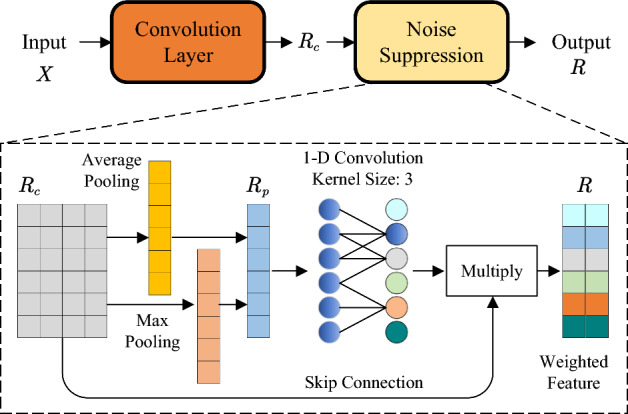
The structure of signal reconstruction module.

To make the original radar echo easy to be processed by the subsequent neural network, we designed a module to reconstruct the radar echo signal in this section. The module expands the original signal and suppresses the noise in the signal. The overall structure of the proposed signal reconstruction module is shown in Fig. 2. As shown in the figure, we use a convolution layer to expand the dimensions of the original input signal *X*. The number of output channels in this layer is greater than the number of input channels. The output expanded feature is denoted by $R_c$.

However, the obtained signal $R_c$ may contain some noise information. It is necessary to suppress the noise in $R_c$ before using deep neural networks to conduct feature encoding. Attention is a promising way to achieve this. It can reconstruct the input features by assigning different weights to different parts of the input features, so that the network can focus on the important parts of the features. Attention has been successfully applied to many pattern recognition tasks in recent years^29–32^. In this paper, we design an attention mechanism for radar signal processing that can filter the noise in the echo. Its structure is shown in Fig. 2.

The fundamental concept behind the signal reconstruction module is to augment the feature dimensionality of the original one-dimensional radar echo sequence, enabling subsequent network structures to effectively extract correlated features. In this module, the convolutional layers are designed with increasing numbers of channels, facilitating the transformation of the input into a more diverse and enriched feature space. Following the convolutional layers, we introduce a noise suppression layer that employs attention mechanisms to redistribute the weights of input features, thereby diminishing the importance of relatively less significant components. This layer incorporates a pooling operation as part of its attention mechanism, effectively compressing the feature dimensionality during weight computation. By incorporating these elements into the signal reconstruction module, we enhance the representation capabilities of the network, enabling it to capture intricate correlations within the radar echoes and facilitate subsequent feature extraction for improved target detection performance.

Specifically, we first use average pooling and maximum pooling to reduce the dimension of the original input signal $R_c$. The pooling operation can enhance the ability of global feature extraction while suppressing noise. Subsequently, the outputs of the two pooling layers will be simply added to obtain $R_p$. Then, fully connected layers are used to capture inter-feature dependencies. As shown in Fig. 2, they can be divided into the early structure and the late structure. The former one is a dimensionality-reduction layer with a reduction ratio $\tau$, and the latter one is a dimensionality-increasing layer that is used to recover the dimension of the pooling result $R_p$. The attention weight is then obtained by the sigmoid function. Multiply these weights by the original input $R_c$, and we can get the weighted features *R*. So far, we have completed the reconstruction of the radar echo signal.

### Self-adaption local augmented LSTM

The radar echo is a context-coupled sequence. One of the most popular models for sequence modeling is Recurrent Neural Network (RNN). In practical applications, a variant of RNN named LSTM^33^ is commonly used to avoid gradient vanishing. The emergence of LSTM solves the problem of gradient vanishing in the standard RNN network. LSTM structure can capture the global dependencies between sequences. For radar target detection, from a global perspective, the network can recognize the overall noise level of the current echo and avoid misjudging the strong sea clutter echo as a sea target. However, global dependency is not enough. At this time, the characteristics of the weak target on the sea surface are easily confused with the echo characteristics of the sea clutter, resulting in misjudgment. Therefore, we should strengthen the ability of LSTM to model local dependencies.

**Figure 3:**
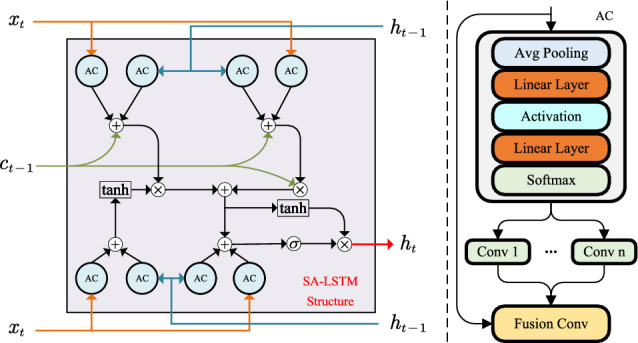
The overall structure of local augmented LSTM.

To achieve the above target, an effective solution is to integrate the convolution layer into LSTM. Sliding convolution kernel can capture local correlation well. However, the network parameters of the traditional CNN structure will be fixed once training is completed, and will be maintained for any input data. If we simply integrate the convolution kernel into the vanilla LSTM cell, it may be difficult to cope with different targets in complex sea scenes due to the limited feature expression ability, so we can not achieve satisfactory detection performance. The possible solution is to enhance the feature representation ability of a single convolution kernel. Inspired by this, we introduce the adaptive convolution, which aggregates multiple convolution kernels by attention mechanism and uses the aggregated kernel to encode input feature^34^.

Based on this idea, we propose self-adaption local augmented LSTM (denoted by SALA-LSTM) for radar target detection in sea clutter. The structure of the proposed SALA-LSTM is shown in Fig. 3. The calculation formulas are as follows:

3 $$\begin{aligned} \begin{aligned} f_z&=\sigma ({\mathscr {F}}_{ac}(R) + {\mathscr {F}}_{ac}(H_{z-1}) + W_{ci}\circ C_{z-1 }+ b_i ) \\ i_z&=\sigma ({\mathscr {F}}_{ac}(R) + {\mathscr {F}}_{ac}(H_{z-1}) + W_{cf}\circ C_{z-1 }+ b_f ) \\ {\tilde{C}}_z&=tanh ({\mathscr {F}}_{ac}(R) + {\mathscr {F}}_{ac}(H_{z-1}) + b_c) \\ C_z&=f_z\circ C_{z-1}+i_z\circ {\tilde{C}}_z \\ o_z&=\sigma ({\mathscr {F}}_{ac}(R) + {\mathscr {F}}_{ac}(H_{z-1})+W_{co}\circ C_z+b_o)\\ H_z&= o_z \circ tanh(C_z) \end{aligned} \end{aligned}$$

where $f_z$ denotes the forget gate, $i_z$ denotes the input gate, and $o_z$ denotes the output gate. $C_z$ and $H_z$ are the hidden states. $\circ$ denotes Hadama product. *W* is the learnable weight matrix and *b* is the bias matrix. ${\mathscr {F}}_{ac}$ represents the adaptive convolution, and its detailed calculation process can be found in Ref.^34^ and Ref.^35^.

### Progressive prediction module

By SALA-LSTM, we can fully consider the local dependence and global dependence to encode the radar echo. The next issue is how to use these encoding features to effectively predict whether the current echo contains sea targets. If only a simple module (such as a fully connected layer) is used to complete the mapping from high-dimensional features to two-class prediction results, much important information will be lost. Therefore, it is necessary to design a sophisticated network structure to achieve this. To this end, we propose a progressive prediction module to extract key information from encoding features layer by layer and finally complete binary prediction.

**Figure 4:**
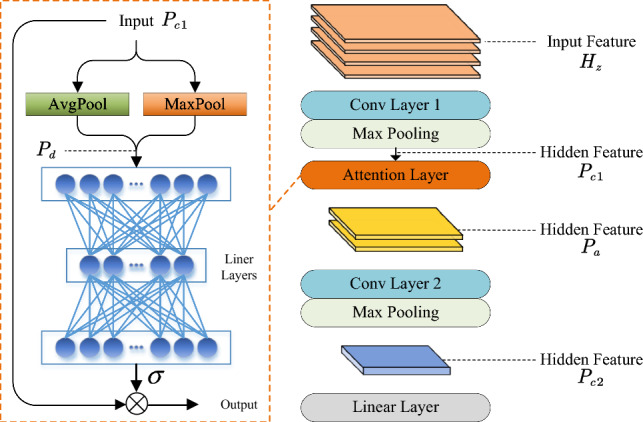
The overall structure of progressive prediction module.

The overall structure of the progressive prediction module is shown in Fig. 4. The basic idea behind the progressive prediction module is to gradually filter the rich correlated features extracted by the preceding network layers, leading to final classification decisions. Within this module, Conv Layer1, Conv Layer2, and the subsequent MaxPooling layers are designed to primarily compress the size of feature maps. By carefully configuring the convolutional kernel sizes and stride lengths in these network structures, the resulting feature maps progressively decrease in size, achieving a filtering effect. This approach of dimensionality reduction provides a “hard” selection mechanism, directly capturing essential features. However, in addition to this strategy, we introduce an attention mechanism within the module to incorporate a more flexible “soft” selection process. The attention mechanism assigns varying weights to the original features, without altering their dimensions. Notably, the max pooling and average pooling operations within the attention mechanism play a crucial role in compressing the feature dimensionality when calculating attention weights. By combining both the “hard” selection through dimension reduction and the “soft” selection through attention mechanisms, the progressive prediction module effectively filters and prioritizes important features, enabling accurate classification decisions in radar target detection tasks.

The specific calculation can be illustrated as follows. Firstly, we use a convolution layer with maximum pooling to extract the local relation of $H_z$:

4 $$\begin{aligned} P_{c1} = MaxPool(W_{c1}H_z+b_{c1}) \end{aligned}$$

where $W_{c1}$ and $b_{c1}$ are the weight parameter and the bias parameter of convolution. Then, we use an attention layer to choose the more important features. The attention weight can be obtained by:

5 $$\begin{aligned}{} &P_d=AvgPooling(P_{c1})+MaxPooling(P_{c1})\\&weight = \sigma \left( W_{f2}\left( W_{f1}P_{d}+b_{f1}\right) +b_{f2}\right) \\ \end{aligned}$$

Then, we multiply the weight and the original feature $P_{c1}$ to get $P_a$. Taking $P_a$ as input, another convolution operation is performed like Eq. (4).

## Experiments and discussion

Using the deployed X-band pulse-compression radar, we conducted sea surface observation experiments. By simple pre-processing, we obtained measured maritime target and sea clutter samples. The detailed introduction of these data and other experimental settings for target detection will be shown in sections “Radar deployment and dataset” and “Experimental settings”. In section “Ablation study”, an ablation study is performed to prove the effectiveness of our proposed detection model. In sections “Detection performance analysis in dataset-S for simulation targets”, “Detection performance analysis in dataset-B for boat targets”, “Detection performance analysis in dataset-A for any maritime targets” and “Detection performance analysis in SDRDSP dataset”, we compare the proposed target detection model with the latest methods on different evaluation datasets.

**Figure 5:**
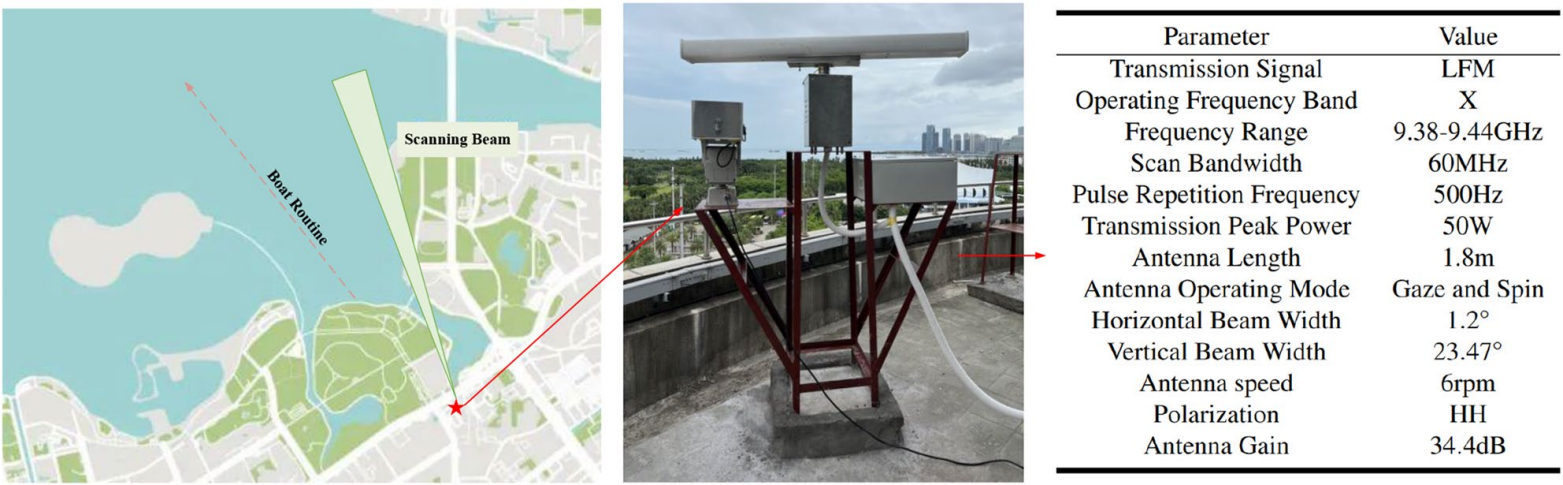
Sea observation scenario of X-band pulse-compression radar. The map is obtained from OpenStreetMap^36^. The data of OpenStreetMap is available under the Open Database License. The copyright and license information can be found in https://www.openstreetmap.org/copyright.

### Radar deployment and dataset

Using the deployed X-band pulse-compression radar, we conducted a series of maritime observation experiments in September and October 2022. The experimental site is on the south coast of China. In the two working modes of staring and scanning, we collected a large number of sea clutter samples. Besides, we collected some maritime targets, such as vessels and boats. After simple data cleaning and tagging, we built a maritime dataset covering different conditions. The dataset will be published in the future to facilitate the researchers in the field of clutter suppression and maritime target detection. The actual deployment location of our radar is shown in Fig. 5.

**Table 1:** Introduction to sample types in different subsets of the dataset.

Dataset	Clutter	Target	Mode
S	Measured	Simulation	Stare
A	Measured	Marine targets within the observation scope	Scan
B	Measured	Fishing boats traveling along a specific route	Scan

The constructed dataset contains three several subsets: Dataset-S, Dataset-B and Dataset-A. The brief description is shown in Table 1. Dataset-S is a large-scale subset. It contains millions of measured sea clutter samples. Additionally, the dataset encompasses five distinct signal-to-noise ratio (SNR) environments, including -10dB, -5dB, 0dB, 5dB, and 10dB. These varying SNR levels serve to simulate diverse noise conditions and ensure a comprehensive evaluation of the method’s detection performance. The simulation strategy employed in this manuscript is briefly described as follows. The transmission signal of our radar system is a linear frequency modulation (LFM) signal, which can be mathematically represented as:

6 $$\begin{aligned} s(t)=A\textrm{rect} \left( \frac{t}{\tau } \right) \exp \left( {j \pi K t^2} \right) , \ K=\frac{B}{\tau } \end{aligned}$$

here *K* represents the linear frequency modulation rate, *B* denotes the frequency bandwidth, and $\tau$ is the pulse duration. According to the radar characteristics, in the simulation conducted in this manuscript, the ideal echo of the transmission signal passing through a target at a certain position is added to the collected sea clutter data with a specific signal-to-noise ratio. The signal-to-noise ratio *SCR* is defined as the ratio of the power of the ideal target to the power of the original sea clutter. The simulated target signal after signal-to-noise ratio correction^22^ is expressed as:

7 $$\begin{aligned} s_c(t)=\sqrt{SCR \cdot P_c }\cdot s(t) \end{aligned}$$

**Figure 6:**
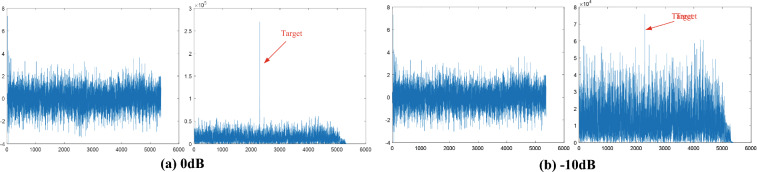
The signal before and after matched filtering.

Subsequently, we randomly superimposed the simulated signal onto a specific location of the original sea clutter signal to complete the entire signal generation process. It should be noted that this manuscript simulates stationary targets on the sea surface and does not consider the influence of Doppler frequency shift. We will perform matched filtering on the synthesized simulation signal, and the echo signal after matched filtering is shown in Fig. 6.

**Figure 7:**
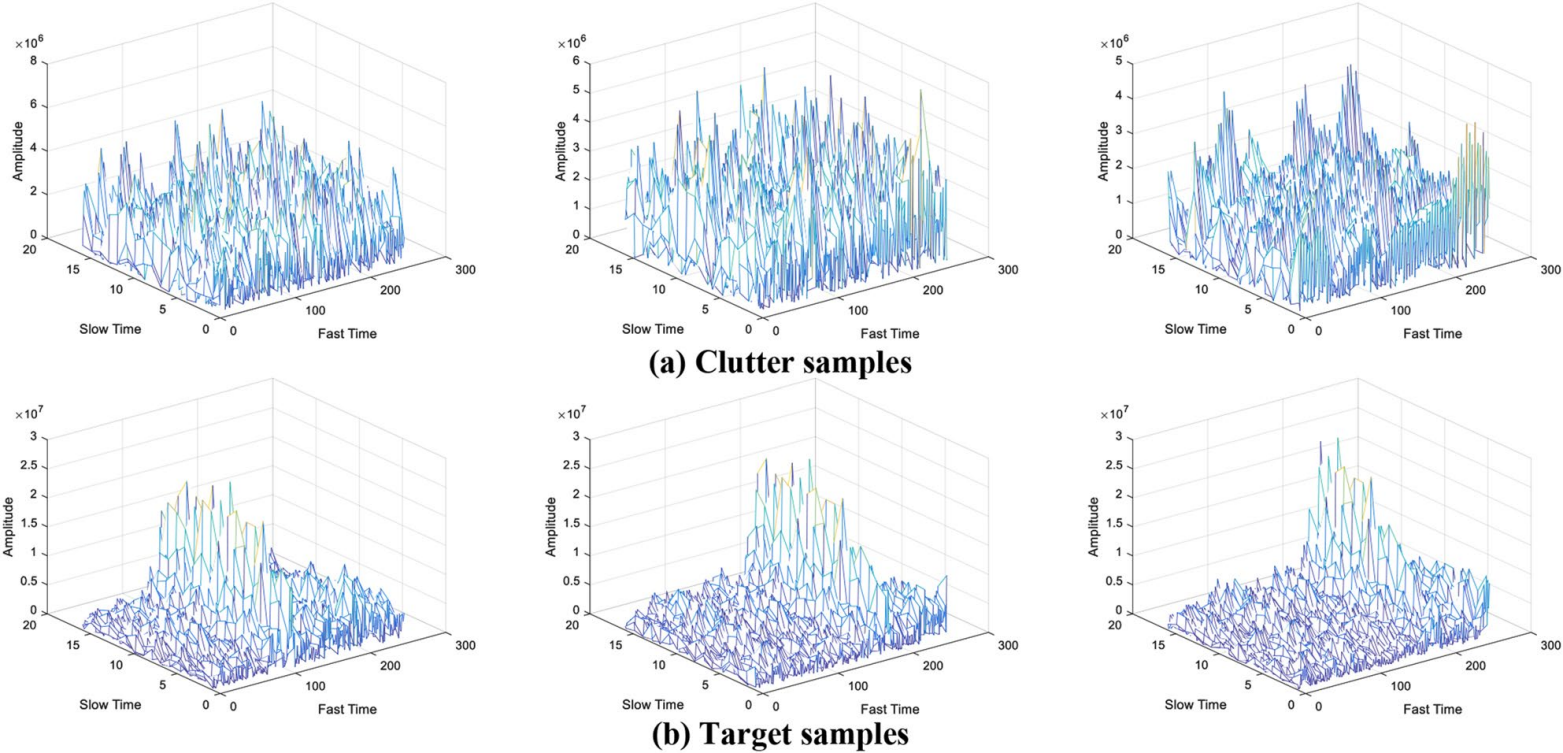
Typical samples of our collected dataset.

The signal-to-clutter ratio (SCR) set in the Dataset-S varies from -10dB to 10dB. Dataset-B contains clutter samples and fishing boat samples. The fishing boat travels along a specific route and berths at a preset position (by GPS). According to the Refs.^37,38^, rain clutter will cause interference to X-band radar. Hence, we collect some target samples on sunny days (Dataset-B1) and rainy days (Dataset-B2). Dataset-A contains clutter samples and all possible maritime target samples (such as boats, vessels, cargo ships, buoys and etc.). We deploy the radar on the shore to collect the echoes of passing maritime targets. By confirming with the real-time data on the Automatic Identification System (AIS) and camera surveillance system, we simply annotated the collected data. The Reflecting Cross Sections (RCS) of these targets are varied. Dataset-A contains six subsets that represent six days of measured data. Typical samples are shown in Fig. 7. For the above three datasets, the numbers of training samples are one hundred thousands (Dataset-S) and one hundred thousands (Dataset-B and Dataset-A). The numbers of testing set samples are one billion (Dataset-S) and two hundred thousands (Dataset-B and Dataset-A).

### Experimental settings

To evaluate the performance of our proposed method, we take CA-CFAR, SKMR-CFAR^39^ and latest deep learning-based methods (denoted by STDN^27^ and DBL^28^) as the comparison methods. The advantage of CFAR is that it does not require supervised training during application, and only simple mathematical calculations can obtain binary detection results. In our experiments, the reference window length and guard cell length are 35 and 55, respectively. This set of parameters was obtained through grid-search experiments. Neural-based detection methods can achieve better detection performance when the amount of training data is sufficient. We use PyTorch to implement the neural model.

The training and testing of this model were completed on the Geforce GTX 2080Ti GPU with 11Gb graphics memory. In the training phase, we use Adam algorithm as the optimizer, and the learning rate is 1e−4. In addition, we choose the binary cross entropy as the loss function. The maximum training epoch of the model is 50, and the batch size is 256. Besides, we present the loss curves of the training and validation procedure according to your advice.

### Ablation study

In this section, we mainly discuss the feature extraction ability of the model variants, so we take classification accuracy as the evaluation metric. The used evaluation dataset is Dataset S. The experimental results are listed in Table 2. LSTM is used as a baseline.

**Table 2:** Ablation experimental results. .

Index	Model variant	Accuracy
$\sharp 1$	Vanilla LSTM + linear layers	0.9915
$\sharp 2$	SALA-LSTM + linear layers	0.9927
$\sharp 3$	Replace SALA-LSTM with Vanilla LSTM	0.9932
$\sharp 4$	Replace PPM with linear layers	0.9975
$\sharp 5$	Replace AC with standard convolution	0.9961
$\sharp 6$	The whole proposed model	**0.9999**

Significant values are in bold.

In $\sharp 1$, we construct a detection network based on vanilla LSTM and linear layers. By comparing the detection accuracies of $\sharp 1$ and $\sharp 2$, it can be concluded that SALA-LSTM can reach a better sequence correlation model performance for radar echoes. Likely, By comparing the detection accuracies of $\sharp 3$ and $\sharp 6$, we can find that if LSTM is directly used to replace SALA-LSTM in the overall network, the detection performance of the network will decrease. The above experiments verify the effectiveness of our proposed SALA-LSTM structure for radar target detection. In $\sharp 4$, we replace the proposed progressive prediction module (PPM) in the overall network with commonly used linear layers. The results show that the detection accuracy of the network will decline. By comparing the results of $\sharp 5$ and $\sharp 6$, we can draw a conclusion that the reasonably designed adaptive convolution module has better detection performance than the traditional CNN structure when being introduced into LSTM.

**Figure 8:**
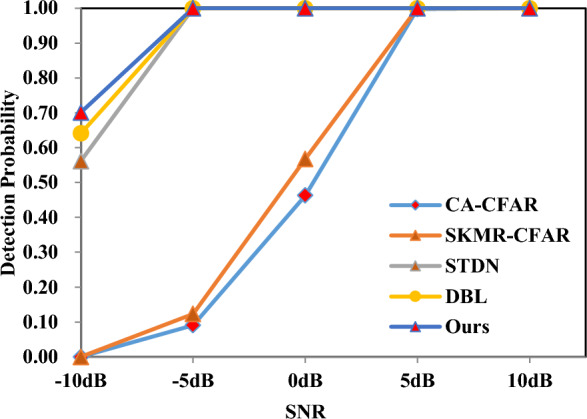
Experimental results in Dataset-S.

### Detection performance analysis in dataset-S for simulation targets

We utilize Dataset-S to fully evaluate the detection probabilities of four methods under different SCR. The experimental results are shown in Fig. 8. Overall, the detection probability of our method is above 70%. When the SCR is relatively high, all the methods can show superior performance. Under 10dB, even CA-CFAR can achieve more than 99% detection probability. When the required SCR is -10dB, our proposed method can reach a detection probability of 70.12%. The sub-optimal method DBL can reach a detection probability of 64.12%. When the SCR is 0dB, CA-CFAR can only reach a detection probability of 46.32%. By contrast, the proposed method in this paper can achieve a detection probability of 99.01%. These results demonstrate that our method has reached the level of practical application and can achieve a high detection probability while maintaining a very low false alarm rate. In general, the detection performance of different methods on the Dataset-S is very high. However, there is a big difference between the target echo received by the radar and the simulated target echo in practice, so we have carried out a detection experiment on the measured target in the following.

### Detection performance analysis in dataset-B for boat targets

In this section, we evaluate the detection performance of different models in measured datasets: Dataset-B1 for boats in sunny days, and Dataset-B2 for boats in rainy days. The experimental results are shown in Table 3. Overall, the neural-based model can reach a better detection probability. In clear weather, fishing boats travel and anchor along the specific route. During this process, we collect radar echo samples synchronously, which constitutes Dataset-B1. When the required false alarm rate is 1e−2, our proposed method can reach a detection probability of 97.78%. The sub-optimal method DBL can reach a value of 95.22%. When the false alarm rate decreases to 5e−3, the detection probability of our method can reach 97.13%, 2.75% higher than that of DBL. When we continue to adjust the prediction threshold, our proposed method can achieve a false detection rate of 1e−3 at the same time with a detection probability of 94.25%. In contrast, under the same experimental settings, CFAR has only a 78.94% probability of correctly identifying whether the current radar echo contains maritime targets.

**Table 3:** Experimental results in dataset-B.

FAR	Detection method				
	CA-CFAR (%)	SKMR-CFAR (%)	STDN (%)	DBL (%)	Ours (%)
B1					
1e−1	94.32	95.09	96.55	98.89	**99.13**
5e−2	90.17	92.32	94.08	98.04	**99.06**
1e−2	87.23	88.95	88.48	95.22	**97.78**
5e−3	82.55	83.67	86.16	94.48	**97.13**
1e−3	78.94	80.15	81.99	91.71	**94.25**
5e−4	70.63	72.13	80.20	88.75	**92.83**
B2					
1e−1	92.32	94.21	96.27	97.25	**98.63**
5e−2	88.17	90.35	93.51	96.10	**98.02**
1e−2	84.23	86.57	87.99	94.18	**96.81**
5e−3	79.55	81.90	85.62	91.74	**96.13**
1e−3	75.94	77.26	80.19	88.34	**92.49**
5e−4	66.63	70.11	78.43	84.65	**88.34**

Significant values are in bold.

The samples of Dataset-B2 are from fishing boats on rainy days. Rain clutter will affect the detection performance of pulse-compression radar^37,38^. Hence, Dataset-B2 is a more difficult detection task compared with Dataset-M2. Even so, our method can achieve 96.81% detection probability when the required false alarm rate is 1e−2, maintaining effective detection. Under the same false alarm rate, the detection probability of CFAR is only 84.23%. When the required false alarm rate decreases 1e−3, our method can still achieve a 92.49% probability of correctly judging whether the current radar echo contains maritime targets.

**Figure 9:**
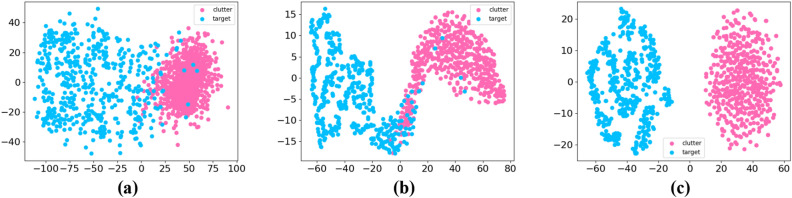
The distribution difference between the maritime target samples and clutter samples by different methods: (**a**) STDN, (**b**) DBL, and (**c**) our method. The figure illustrates the distribution differences between maritime target samples and clutter samples. It is noteworthy that our proposed method achieves a more pronounced separation effect compared to DBL and STDN.

In our study, we conducted feature visualization experiments to gain insights into the performance of different neural-based methods. For this purpose, we employed the t-SNE algorithm^40^ to visualize the output features generated by these methods. The results are presented in Fig. 9. From the visualization in Fig. 9, it is apparent that there exist notable distribution differences between the maritime target samples and clutter samples. However, when comparing the output features of the two neural methods, DBL and STDN, we observe some overlap in their distributions. On the other hand, the distribution of the output features produced by our method demonstrates significant dissimilarity. This finding suggests that our proposed neural model effectively extracts distinctive features from radar echoes, resulting in a greater separability between the target and clutter samples. In conclusion, the effectiveness of our proposed neural model in extracting features from radar echoes was verified through feature visualization experiments. These findings provide guidance for further enhancing target detection and recognition algorithms, with the potential to improve the performance and reliability of radar systems in practical applications.

**Table 4:** Detection experimental results in dataset-A.

Set	Detection method				
	CA-CFAR (%)	SKMR-CFAR (%)	STDN (%)	DBL (%)	Ours (%)
FAR					
D1	11.93	8.25	0.82	1.81	**0.12**
D2	6.99	3.26	0.49	1.00	**0.04**
D3	9.10	5.77	0.78	1.66	**0.47**
D4	2.42	1.92	0.81	1.69	**0.32**
D5	2.44	1.05	0.70	0.97	**0.25**
D6	2.81	1.36	**0.50**	0.97	0.77
Avg	5.95	3.60	0.68	1.35	**0.33**
DP					
D1	96.86	97.99	91.89	93.44	**98.92**
D2	95.51	97.36	91.61	93.33	**99.96**
D3	88.65	92.57	96.45	95.44	**98.79**
D4	90.79	93.09	97.70	97.97	**99.79**
D5	83.66	87.71	93.91	94.12	**94.58**
D6	92.86	94.25	96.01	96.38	**99.87**
Avg	91.39	94.93	93.10	95.11	**98.65**

Significant values are in bold.

### Detection performance analysis in dataset-A for any maritime targets

**Figure 10:**
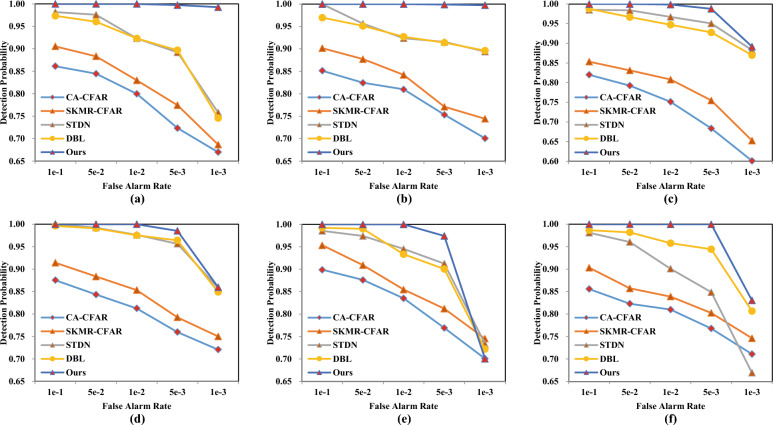
ROC curve in Dataset-A. Different subfigures represent results on different subsets.

The samples of Dataset-A are from all possible targets passing on the sea surface. The RCS of these targets are varied. Dataset-A contains six subsets (D1$\sim$D6) that represent six days of measured echo data. The experimental results are listed in Table 4. Then, we adjust the classification threshold and the obtained receiver operating characteristic (ROC) curve are shown in Fig. 10. From these two sets of data, our method achieves optimal detection performance. When the required false alarm rate is 1e−2, our proposed method can reach a detection probability of 99%. The sub-optimal method DBL can reach a value of 97.52% in subset D4 and 92.31% in subset D1. When we continue to adjust the prediction threshold, our proposed method can achieve a false detection rate of 1e−3 at the same time with a detection probability of 99.28% in subset D1. In contrast, under the same experimental settings, STDN has only an 75.82% probability of correctly identifying whether the current radar echo contains maritime targets.

Dataset S is simulated datasets that do not consider many noise interference that may affect system performance. They represent relatively ideal detection scenarios, and therefore our proposed method can achieve close to 100% detection probability on the dataset. However, real-world detection scenarios differ significantly from simulation scenarios. In real-world scenarios, radar target detection algorithms often struggle to achieve near-perfect accuracy due to interference from different complex sea conditions and the varying radar reflection characteristics of the maritime targets. The experimental results in our paper also confirm this conclusion. Therefore, we believe that improving the detection performance of radar target detection algorithms in practical application scenarios is an important direction for future research.

### Detection performance analysis in SDRDSP dataset

**Figure 11:**
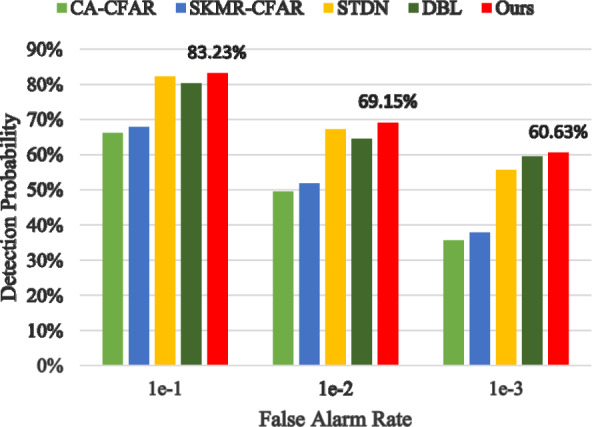
Detection performance in SDRSDP dataset.

In addition to our constructed dataset, we further evaluate the detection performance of our proposed method by integrating other publicly available datasets for comprehensive verification. With the continuous advancement of radar technology, the Information Fusion Institute at the Naval Aviation University has introduced the Sea-detecting Radar Data-sharing Program (SDRDSP) to meet the growing demand for radar data. In the sea clutter and target detection experiments conducted in 2020, the radar was situated at an elevation of 80 meters on a bathing beach in Yantai City. To further evaluate the effectiveness of our proposed detector, we have added comparative experiments and tested the detection performance on SDRDSP dataset^41^ in this section. The selected file “20210106160919_1” is used for training, and “20210106160919_2” is used for testing. The experimental results are shown in Fig. 11.

**Figure 12:**
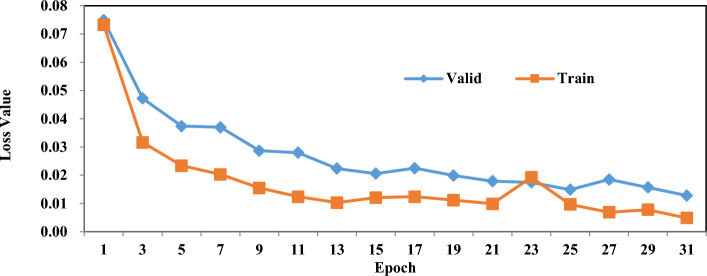
The loss terms change in the process of model training for SDRSDP Dataset.

Furthermore, we provide a detailed analysis of the loss term (binary cross entropy) as it evolves across different stages of network training, as illustrated in Figure 12. The data presented in the figure demonstrates a steady decrease in the loss term on the training set over time, indicating that our network effectively learns and adapts to the given data. This progressive reduction in the loss term signifies the optimization process, where the network gradually minimizes the discrepancies between its predicted outputs and the ground truth labels. The decreasing trend observed in the loss term is an encouraging indication of the network’s ability to accurately classify and distinguish between different classes or categories. As the iterations progress, the loss continues to decline, suggesting that the network is continuously refining its internal representations and updating its parameters to better capture the underlying patterns and relationships within the data. Ultimately, the loss term converges towards zero, indicating that the network has successfully learned the intricate features and nuances required for accurate predictions.

## Conclusion

Radar detection of maritime targets plays an increasingly important role in marine environment monitoring. To better model the sequence correlation of radar echoes, we propose a SALA-LSTM structure. Based on SALA-LSTM and other neural structures (such as signal reconstruction module and progressive prediction module), we propose a radar target detection network. In the future, we will utilize the deployed X-band pulse-compression radar to obtain more maritime target samples to further improve the performance of our method.

## Data Availability

The datasets used during the current study available from the corresponding author on reasonable request.
